# Deaths in Philadelphia from Sept. 21st to Oct. 19th, 1850

**Published:** 1850-11

**Authors:** James Aitken Meigs

**Affiliations:** Student of Medicine


*Deaths in Philadelphia from Sept.* 21*st to Oct.* 19*th*, 1850. Reported by Mr. JAMES AITKEN MEIGS, Student of Medicine.

| Diseases. | Ad'ts | Chil. | Diseases. | Ad'ts | Chil. |
|---|---|---|---|---|---|
| Abscess, . . . | 1 | 0 | Fever, puerperal, . . | 3 | 0 |
| " of lungs, . . | 1 | 1 | " remittent, . . | 6 | 0 |
| " of perineum, . | 0 | 1 | " scarlet, . . | 0 | 10 |
| Apoplexy, . . . | 7 | 0 | " typhoid, . . | 12 | 4 |
| Asthma, . . . . | 1 | 0 | " typhus, . . | 9 | 1 |
| Burns, . . . | 2 | 1 | Fistula, . . . . | 1 | 0 |
| Cancer of face, . . | 1 | 0 | Fracture, . . . | 1 | 0 |
| " stomach, . | 2 | 0 | " of leg, . . | 1 | 0 |
| " uterus, . . | 1 | 0 | " skull, . . | 0 | 1 |
| Carditis, . . . | 0 | 1 | Gangrænopsis, . . | 0 | 1 |
| Casualties, . . . | 5 | 3 | Gangrene of intestines, . | 0 | 1 |
| Cholera infantum, . . | 0 | 11 | Hæmoptysis, . . . | 1 | 0 |
| " morbus, . . | 1 | 0 | Hemorrhage . . . | 1 | 0 |
| Congestion of brain, . | 5 | 3 | " uterine, . | 1 | 0 |
| " liver, . | 0 | 1 | Hernia, strangulated, . | 1 | 0 |
| " lungs, . | 3 | 2 | Inflammation of brain, . | 2 | 7 |
| Convulsions, . . . | 1 | 33 | " breast, . . | 0 | 3 |
| " puerperal, . | 0 | 1 | " bronchi, . . | 2 | 12 |
| Croup, . . . . | 0 | 6 | " liver, . . | 1 | 3 |
| Cyanosis, . . . | 0 | 2 | " lungs, . . | 8 | 7 |
| Debility, general, . . | 9 | 12 | " parotid gland, . | 0 | 1 |
| Diabetes, . . . | 1 | 0 | " peritoneum, . | 3 | 4 |
| Diarrhœa, . . . | 5 | 10 | " pleura, . . | 1 | 0 |
| Disease of brain, . . | 2 | 0 | " stom. & bowels, | 11 | 5 |
| " heart, . . | 6 | 1 | Inanition, . . . | 1 | 3 |
| " kidneys, . | 2 | 0 | Intemperance, . . | 2 | 0 |
| " liver, . . | 2 | 0 | Intussusception, . . | 2 | 0 |
| " lungs, . . | 1 | 2 | Jaundice, . . . | 0 | 1 |
| " spine, . . | 0 | 1 | Malænia, . . . | 1 | 0 |
| " stomach and bowels, | 2 | 1 | Malformation, . . | 0 | 4 |
| Dropsy, . . . | 8 | 1 | Mania-a-potu, . . | 9 | 0 |
| " abdominal, . | 3 | 1 | Marasmus, . . . | 2 | 23 |
| " of breast, . . | 2 | 1 | Measles, . . . | 0 | 1 |
| " of head, . . | 0 | 13 | Mortification, . . . | 1 | 0 |
| Drowned, . . . | 3 | 2 | Œdema glottidis, . . | 1 | 0 |
| Dysentery, . . . | 26 | 16 | Old age, . . . . | 17 | 0 |
| Effusion on brain, . . | 1 | 5 | Palsy, . . . . | 4 | 0 |
| " lungs, . . | 1 | 0 | Perforation of stomach, . | 1 | 0 |
| Emphysema, pulmonary, | 1 | 0 | Pertussis, . . . | 0 | 9 |
| Enlargement of liver, . | 1 | 0 | Phthisis pulmonalis, . | 66 | 2 |
| Epilepsy, . . . | 1 | 0 | Poisoning, . . . | 0 | 1 |
| Erysipelas, . . . | 2 | 2 | Scirrhus, . . . | 1 | 0 |
| Exhaustion, . . . | 1 | 0 | " of stom. and liver, | 1 | 0 |
| Fever, . . . . | 0 | 1 | Softening of heart, . | 1 | 0 |
| " bilious, . . | 3 | 1 | " spinal marrow, | 1 | 0 |
| " cerebral, . . | 1 | 0 | " stomach, . | 0 | 1 |
| " congestive, . . | 3 | 1 | Spina bifida, . . . | 0 | 1 |
| " hectic, . . | 0 | 1 | Still born, . . . | 0 | 32 |
| " intermittent, . | 1 | 0 | Tabes mesenterica, . | 0 | 2 |

| Diseases. | Ad'ts | Chil. | Diseases. | Ad'ts | Chil. |
|---|---|---|---|---|---|
| Teething, . . . . | 0 | 1 | Ulceration of throat, . | 1 | 0 |
| Tetanus, . . . | 1 | 1 | Unknown, . . . . | 2 | 2 |
| Tuberculosis, . . . . | 0 | 1 | Varicella, . . . . | 2 | 1 |
| Ulceration of small intest. | 1 | 1 | Violence, . . . . | 1 | 0 |
| | | | | 303 | 291 |

Total, . . . . . . . . . . . . . . 594

Of the foregoing the ages were as follows:—

| | | | | |
|---|---|---|---|---|
| Under | 1 | year, | - | 146 |
| From | 1 | to - | 2, | 62 |
| | 2 | - - | 5, | 40 |
| | 5 | - - | 10, | 24 |
| | 10 | - - | 15, | 7 |
| | 15 | - - | 20, | 12 |
| | 20 | - - | 30, | 67 |
| | 30 | - - | 40, | 74 |
| | 40 | - - | 50, | 56 |
| | 50 | - - | 60, | 41 |
| | 60 | - - | 70, | 29 |
| | 70 | - - | 80, | 20 |
| | 80 | - - | 90, | 13 |
| | 90 | - - | 100, | 3 |
| | | | | 594 |

Included in this number, are 56 from the Almshouse, 20 from the surrounding country, and 38 people of color.

